# Substituting Far-Red for Traditionally Defined Photosynthetic Photons Results in Equal Canopy Quantum Yield for CO_2_ Fixation and Increased Photon Capture During Long-Term Studies: Implications for Re-Defining PAR

**DOI:** 10.3389/fpls.2020.581156

**Published:** 2020-09-11

**Authors:** Shuyang Zhen, Bruce Bugbee

**Affiliations:** Crop Physiology Laboratory, Department of Plants Soils and Climate, Utah State University, Logan, UT, United States

**Keywords:** canopy photosynthesis, carbon use efficiency, far-red photons, quantum yield, phytochrome equilibrium, radiation capture

## Abstract

Far-red photons regulate shade avoidance responses and can have powerful effects on plant morphology and radiation capture. Recent studies have shown that far-red photons (700 to 750 nm) efficiently drive photosynthesis when added to traditionally defined photosynthetic photons (400–700 nm). But the long-term effects of far-red photons on canopy quantum yield have not yet been determined. We grew lettuce in a four-chamber, steady-state canopy gas-exchange system to separately quantify canopy photon capture, quantum yield for CO_2_ fixation, and carbon use efficiency. These measurements facilitate a mechanistic understanding of the effect of far-red photons on the components of plant growth. Day-time photosynthesis and night-time respiration of lettuce canopies were continuously monitored from seedling to harvest in five replicate studies. Plants were grown under a background of either red/blue or white light, each background with or without 15% (50 μmol m^−2^ s^−1^) of far-red photons substituting for photons between 400 and 700 nm. All four treatments contained 31.5% blue photons, and an equal total photon flux from 400 to 750 nm of 350 μmol m^−2^ s^−1^. Both treatments with far-red photons had higher canopy photon capture, increased daily carbon gain (net photosynthesis minus respiration at night), and 29 to 31% more biomass than control treatments. Canopy quantum yield was similar among treatments (0.057 ± 0.002 mol of CO_2_ fixed in gross photosynthesis per mole of absorbed photons integrated over 400 to 750 nm). Carbon use efficiency (daily carbon gain/gross photosynthesis) was also similar for mature plants (0.61 ± 0.02). Photosynthesis increased linearly with increasing photon capture and had a common slope among all four treatments, which demonstrates that the faster growth with far-red photon substitution was caused by enhanced photon capture through increased leaf expansion. The equivalent canopy quantum yield among treatments indicates that the absorbed far-red photons were equally efficient for photosynthesis when acting synergistically with the 400–700 nm photons.

## Introduction

Plants capture light as fuel for photosynthesis and perceive fluctuations in their radiation environments as signals that trigger changes in plant shape, biochemical composition, developmental stages and resource allocation (Kendrick and Kronenberg, 1994). These adaptive responses are mainly directed by three aspects of light: spectral quality, quantity, and duration. Among the photoreceptors, the red/far-red absorbing phytochromes are of crucial importance in sensing vegetation shade and eliciting shade avoidance responses that often maximize plant growth and fitness in crowded stands (Smith, 1982; Schmitt et al., 1995). Green leaves absorb efficiently in the photosynthetically active region of the spectrum (PAR; 400 to 700 nm) but strongly transmit and reflect longer wavelength far-red photons above 700 nm (Woolley, 1971; Kasperbauer, 1987). This selective attenuation causes the red (R) to far-red (FR) ratio to decrease in forest understories or within dense canopies compared to unfiltered sunlight (Federer and Tanner, 1966; Holmes and Smith, 1977; Casal, 2013). Even before plants are directly shaded, small quantities of additional far-red reflected by neighboring plants are perceived *via* phytochromes as an early warning signal of competition and induce rapid stem elongation in shade-avoiding seedlings (Ballaré et al., 1987). Increased stem (and petiole) extension growth is a most prominent shade avoidance response (Smith and Whitelam, 1997; Franklin, 2008).

Additionally, reduced branching and tillering, lower leaf to stem dry mass ratio, smaller proportion of biomass allocation to the roots, hyponastic (upward bending) leaves with reduced chlorophyll (chl) content, and earlier flowering are among the most frequently observed responses to low R:FR ratios in shade-avoiding species adapted to open habitats (Morgan and Smith, 1979; Kasperbauer, 1987; Halliday et al., 1994; Smith and Whitelam, 1997; Casal, 2012). Those shade-avoidance responses are also induced in agronomic crops under high density monocultures and can cause crops to be more prone to drought and lodging and negatively affect yield, thus are often considered undesirable (Morgan et al., 2002).

Unlike the shade-avoiding species, some plants tolerate shade without showing a strong, or any phytochrome-mediated stem extension growth. Instead, those shade-tolerant species maximize radiation capture through leaf expansion, which is accompanied with thinner leaves and increased fractional biomass allocation to leaves (Gommers et al., 2013). Photosynthetic efficiency is also optimized under shade light environments (*i.e.* reduced PAR and relatively enriched FR) *via* adaptive changes in the structure, composition and function of thylakoid membranes, *e.g.* increased PSII:PSI ratio and lower chl a:b ratio (Anderson et al., 1988; Chow et al., 1990a; Valladares and Niinemets, 2008). Both shade-avoiding and -tolerant species perceive shade using the same family of photoreceptors (*i.e.* phytochromes) and share a number of overlapping responses (*e.g.* reductions in leaf thickness). The underlying regulatory pathways that lead to the contrasting phenotypic responses between two strategies remain unclear (see Gommers et al., 2013 for an in-depth discussion of potential regulators of shade tolerance responses).

Because crop productivity exhibits a strong linear correlation with radiation intercepted by canopies (Monteith, 1977; Gifford et al., 1984; Beadle and Long, 1985), increased leaf expansion and accelerated canopy closure are desirable traits, especially in annual crops. Recent advances in light emitting diode (LED) technology enable precise control of spectral quality in controlled environment crop production (Massa et al., 2008; Kusuma et al., 2020). Perhaps the most powerful effect is the simulation of shade through FR supplementation, often without reducing light intensity in the PAR region. This change has been reported to promote leaf expansion in various leafy greens, fruiting vegetables, and ornamental species, which increases growth primarily through increased radiation capture (Casal et al., 1987; Goins et al., 2001; Li and Kubota, 2009; Stutte et al., 2009; Park and Runkle, 2017; Kalaitzoglou et al., 2019).

One important difference between simulated shade and natural vegetation shade is that natural shade significantly reduces total photon flux (Casal, 2012). Leaf expansion induced by far-red photons may interact with photon flux as it is partly dependent on supply of photosynthates (Dale, 1988). Heraut-Bron et al. (1999) observed that lowering R:FR ratio induced leaf expansion of white clover when plants were grown under high photosynthetic photon flux density (PPFD) of 320 μmol m^−2^ s^−1^ but not under low PPFD of 110 μmol m^−2^ s^−1^. Leaf expansion induced by far-red supplementation in most simulated shade studies have been conducted under medium to high light conditions. In order to promote beneficial leaf expansion with a high fraction of FR photons, an adequate photon flux in the PAR region is likely needed.

While the photomorphogenic effects of FR photons are largely well characterized, far-red photons (*λ* > 700 nm) are generally thought to be ineffective for photosynthesis due to their poor photosynthetic efficiency when applied alone (Emerson and Lewis, 1943; McCree, 1972; Inada, 1976). Far-red photons are thus excluded from the current definition of photosynthetic photons. However, pioneering research by Emerson and co-workers found that photosynthetic rate is enhanced under simultaneous illumination of short- and long-wavelength (*λ* >685 nm) photons (Emerson et al., 1957; Emerson and Rabinowitch, 1960). This phenomenon, known as the Emerson enhancement effect, indicates that the effect of different wavelength photons on photosynthesis may not be simply additive. This finding contributed to the subsequent identification of two photosystems with different absorption properties (Hill and Bendall, 1960; Duysens and Amesz, 1962). More recent work demonstrates that most of the shorter wavelength photons from 400 to 680 nm over-excite photosystem II (PSII), while longer wavelength far-red photons preferentially excite photosystem I (PSI) (Evans, 1987; Hogewoning et al., 2012; Laisk et al., 2014; Zhen et al., 2018). When combining far-red with shorter wavelength photons, the balance of excitation distribution between PSII and PSI is restored, thus leading to increased leaf photochemical efficiency (Zhen and van Iersel, 2017) and photosynthetic rate (Hogewoning et al., 2012; Murakami et al., 2018). Scaling up from leaf to canopy level, Zhen and Bugbee (2020) found that far-red photons (700–750 nm) are equally efficient at driving canopy photosynthesis when added to 400 to 700 nm photons in 14 diverse crop species. These recent findings warrant re-consideration of the photosynthetic value of far-red photons.

Following canopy photon capture and photosynthetic efficiency, the third determinant of daily carbon gain and productivity is the conversion efficiency of carbon fixed in gross photosynthesis into biomass, *i.e.* carbon use efficiency (Gifford et al., 1984; Bugbee and Monje, 1992). The loss of carbon to respiration at whole-plant/canopy level can be more than 50% of gross photosynthesis (Amthor, 1989). To our knowledge, the effects of far-red photons on canopy quantum yield (moles of CO_2_ fixed in gross photosynthesis per mole of absorbed photons; a measure of photosynthetic photon use efficiency) and carbon use efficiency (CUE = ratio of C incorporated into biomass to C fixed in gross photosynthesis) during long-term crop cultivation have not been studied. Most previous studies have added far-red photons as a supplement, which complicates interpretation of the results because total photon flux from 400 to 750 nm is not constant.

Our objective was to quantify the effects of far-red substitution for 400–700 nm photons on radiation capture, canopy quantum yield, carbon use efficiency, and biomass allocation of a model crop lettuce. This quantitative approach can provide a mechanistic understanding of the effect of far-red photons on plant growth. Continuous measurement of canopy quantum yield for CO_2_ fixation can provide additional evidence for changing the definition of photosynthetic active radiation to include far-red photons (700–750 nm).

## Materials and Methods

### Plant Materials

Lettuce (*Lactuca sativa* cv. Waldmann’s Dark Green) was seeded in 1.7 L containers filled with calcined clay (Greens Grade; Profile, Buffalo Grove, IL) in a glass-covered greenhouse and then moved into a multi-chamber gas exchange system two to four days after emergence. The calcined clay substrate was rinsed with de-ionized water prior to use as described by Adams et al. (2014) and soaked with a hydroponic nutrient solution (Utah hydroponic refill solutions for dicots with FeCl_3_ and iron-chelating agent EDDHA doubled in concentration; USU Crop Physiology Laboratory, 2018). The pH of the hydroponic solution was adjusted to 5.8 with nitric acid and the electric conductivity of the solution was 1 ± 0.1 mS/cm. This low pH minimized bicarbonate effects on CO_2_ fluxes from the root-zone (van Iersel and Bugbee, 2000). Seedlings were selected for uniformity and thinned to one plant per container. Five replicate studies were conducted.

### Light Treatments and Growing Conditions

**Figure 1:**
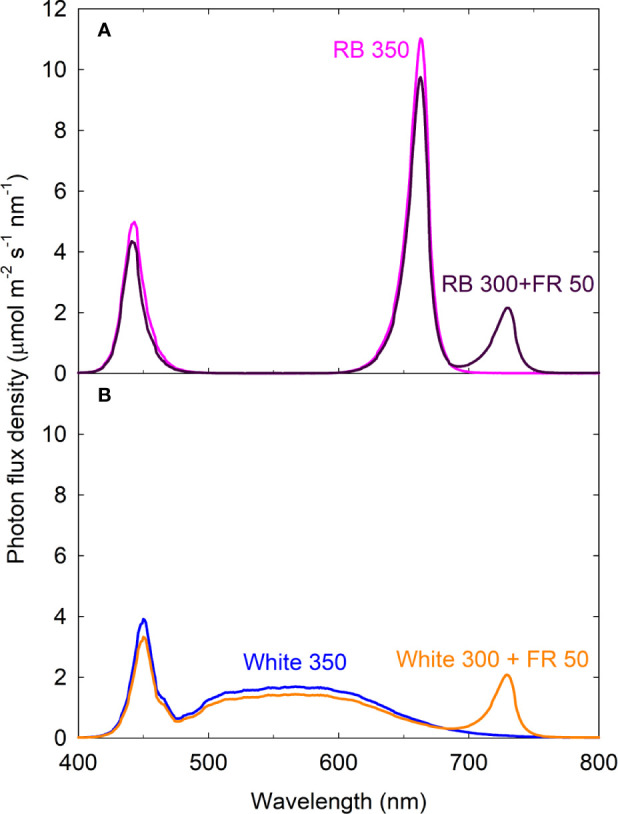
Spectral distributions of four light treatments composed of red/blue (RB; peaks at 443 and 663 nm), white (peak at 450 nm with secondary peak at 567 nm), and far-red (FR; peak at 730 nm) photons. The numbers following each type of light (*e.g.* RB 350) indicate photon flux density in μmol m^−2^ s^−1^. All four treatments had equal total photon flux of 350 μmol m^−2^ s^−1^ (400 to 750 nm) and 31.5% blue photons.

Sixteen uniform seedlings were moved into a steady-state gas exchange system with four acrylic chambers (100 L/chamber with four plants per chamber; 36 cm × 47 cm × 59 cm; w × l × h), similar to the multi-chamber gas exchange system described by van Iersel and Bugbee (2000). Each chamber consisted of one spectral treatment, with cool white, red, blue, and far-red LEDs (Ray 22; Fluence Bioengineering, Austin, TX, USA) placed on top of the chambers. The four light treatments were: white 350, red/blue (RB) 350, white 300 + far-red (FR) 50, and RB 300 + FR 50 (see **Figure 1** for spectral distributions). The numbers following each type of light (*e.g.* RB 300) indicate the average photon flux density in μmol m^−2^ s^−1^ [±1 μmol m^−2^ s^−1^; measured at 13 locations across the chamber floor area of 0.17 m^2^ using a spectroradiometer (SS-110; Apogee Instruments, Logan, UT, USA)]. The blue (400–500 nm): green (501–600 nm): red (601–700 nm) ratio was in 31.5:45.2:23.3 in white 350 and 31.5:0.1:68.4 in RB 350. Phytochrome photoequilibrium [PPE, an indicator of the relative amount of active phytochrome in the far-red-absorbing P_fr_ form] under each light spectrum was calculated using the phytochrome photoconversion coefficients by Sager et al. (1988) (**Table 1**).

**Table 1:** Effect of Spectral Quality on Growth, Morphology, and Chlorophyll Content of Lettuce.

	Spectra and photon flux density			
	RB 350	RB 300 + FR 50	White 350	White 300 + FR 50
(PPE)	(0.87)	(0.82)	(0.84)	(0.70)
Shoot fresh mass (g)	130^b^	179^a^	129^b^	167^a^
Shoot dry mass (g)	8.6^b^	11.3^a^	8.5^b^	11.2^a^
Shoot dry/fresh mass (%)	6.7^a^	6.4^a^	6.6^a^	6.8^a^
Root dry mass (g)	1.61^a^	1.85^a^	1.61^a^	1.95^a^
% root	16.1^a^	14.7^a^	16.6^a^	15.3^a^
Leaf area (cm^2^)	3091^b^	4423^a^	3111^b^	4228^a^
Leaf mass per area (g m^−2^)	28.2^a^	25.5^c^	27.5^ab^	26.5^bc^
Chl content (μmol m^−2^)	272^a^	218^b^	267^a^	213^b^
Leaf photon absorption	0.927	0.798	0.838	0.686

PPE, Phytochrome photoequilibrium; RB, red and blue; FR, far-red. The number following each type of light (e.g. RB 350) indicates photon flux density in μmol m^−2^ s^−1^. Biomass and leaf area were expressed on a per chamber basis (i.e. the sum of four plants). Data represent means from five replicate studies with different letters indicate significant differences between means (P ≤ 0.05; n = 5). Leaf photon absorption of incident photons under each spectral treatment (mol _absorbed_ mol^−1^_incident_) was integrated from 400 to 750 nm.

Chamber walls were lined with highly reflective Mylar to eliminate side lighting and increase light uniformity. The reflective walls also help to simulate light environment of a canopy because they reflect FR photons similar to neighboring plants. Two of the treatments had FR photons (700–750 nm) substituting for ~15% of the conventionally defined white or RB photosynthetic photons. All four treatments contained 31.5% blue photons and equal total photon flux of 350 μmol m^−2^ s^−1^ from 400 to 750 nm. Photoperiod was 14/10 h light/dark. Daily light integral was 17.64 mol m^−2^ d^−1^.

Plants were grown under elevated CO_2_, with the CO_2_ concentration [CO_2_] in the pre-chamber air streams enriched to 800 μmol mol^−1^. CO_2_ enrichment is a common practice in controlled environment crop production. Controlling CO_2_ to a constant elevated level also reduces noises in the gas exchange measurements caused by un-steady [CO_2_] in the ambient air in urban areas. Air flow rate through the chambers was gradually increased from 11 to 37 mmol s^−1^ (15 to 50 standard L min^−1^) from seedling to mature plant stage. Air inside the chambers was mixed with fans (793 L min^−1^; Model A36-B10A-15T2-000, Globe Motors). The [CO_2_] inside the chambers ranged from 740 (with mature plants) to 800 μmol mol^−1^ (seedlings) during the light periods and from 800 (seedlings) to 820 μmol mol^−1^ (mature plants) during the dark respiration periods. Flow rates through the chambers were adjusted depending the size and photosynthetic rate of canopies to maintain the [CO_2_] inside each chamber within 5 μmol mol^−1^ of each other. Chamber air temperature was controlled with resistance heaters and maintained constant at 25 ± 0.1°C day/night. Plants were watered daily with the hydroponic nutrient solution described above (Bugbee, 2004).

### Canopy Gas Exchange Measurements and Calculations

The pre- and post-chamber air streams were sampled every second using two infra-red gas analyzers (reference and differential IRGA; LI-6252; LI-COR, Lincoln, NE). Each chamber was sampled for 30 s, and then the tubing that connected the chambers to the differential IRGA was purged for 40 s before the next chamber was sampled. Canopy gas exchange rate [net photosynthetic rate during the light period (P_net, light_) and dark respiration rate (R_dark_; negative values) in μmol_CO2_ m^−2^_ground area_ s^−1^] was calculated from the mass flow rate and delta [CO_2_] between the pre- and post-chamber air streams and recorded using a datalogger (CR1000; Campbell Scientific, Logan, UT, USA) throughtout the entire course of the study.

To eliminate the mole fraction dilution of CO_2_ analysis by water vapor, the air streams sampled by the IRGAs were first passed through nafion dryers (Perma Pure, Lakewood, NJ, USA) and then columns of magnesium perchlorate to completely remove water vapor.

To determine the photosynthetic value of FR photons, far-red LEDs in the two FR substitution treatments were turned off for 30 to 40 min during the days prior to harvest.

P_net, light_ and R_dark_ was averaged over the 14 h of light period and 10 h of dark period respectively, on a daily basis. The averaged values (in units of mol_CO2_ m^−2^_ground area_ h^−1^) were used in the subsequent calculations of gas exchange parameters (Eqs 1–3).

Canopy gross photosynthesis (P_gross_; g d^−1^) was calculated as:

(1) $$P_{\text{gross}}=(P_{\text{net, light}}+|R_{\text{dark}}|)\;\times \;14\;h\times 0.17\;m^{2}\times 30\;g\;{\text{mol}}^{-1}$$

Where |R_dark_| was the absolute value of dark respiration. 14 was the light period in hours, and 0.17 was the chamber ground area in m^2^. 30 represents grams dry mass per mole of CO_2_ assimilated, assuming a carbon content of 0.4 g g^−1^ in plant tissues. This calculation of P_gross_ also assumes that respiration rate was similar in the light and dark. There is ongoing research and discussion on whether respiration rate in the light is similar, higher, or lower than respiration in the dark (Sharp et al., 1984; Atkin et al., 1998; Frantz et al., 2004 and citations therein). We assumed that respiration rate in light and dark was the same, which is the standard assumption in canopy photosynthesis research (van Iersel, 2003).

Daily carbon gain (DCG; a measure of canopy growth rate in g d^−1^) was calculated as:

(2) $$\text{DCG}=(P_{\text{net, light}}\times 14\;\text{h -}|R_{\text{dark}}|\;\times \;10\;\text{h)}\times {0.17\;m}^{2}\times {30\text{ g mol}}^{-1}$$

Where 14 was the light period in hours, and 10 was the dark period in hours.

Carbon use efficiency (CUE; the ratio of daily net carbon gain to the total amount of carbon fixed in gross photosynthesis) was calculated from Eqs (1) and (2) as:

(3) $${\text{CUE = DCG/P}}_{\text{gross}}$$

### Imaging of Canopy Ground Cover

Plants were taken out of the gas exchange chambers for 2 to 3 min daily for top down photos of the canopies, taken with a digital camera placed 130 cm above the plant base. Images were analyzed using a Python program, available as open source at https://github.com/jakobottar/green-pixel-analysis. The fraction of canopy ground cover was calculated as the ratio of green pixels (plant tissues) to the total pixel count of an area equal to the chamber ground area as described by Klassen et al. (2004).

### Growth Parameters and Chlorophyll Content

Plants were destructively harvested 17 to 20 days after transferred into the gas exchange chambers, when the largest canopies were near canopy closure. Shoot fresh weight and total leaf number of all four plants in each spectral treatment were recorded. Leaf chlorophyll content (μmol m^−2^) was measured on five representative leaves per plant and averaged over four plants per treatment (MC-100 chlorophyll meter; Apogee Instruments, Logan, UT). Total leaf area per chamber was measured using a leaf area meter (LI-3000; LI-COR, Lincoln, NE, USA). Shoots and roots were oven-dried to a constant mass (at least 48 h at 80°C). Root percent mass was calculated as root dry mass/(total shoot and root dry mass). Leaf mass per area (g m^−2^), a measure of leaf thickness, was calculated as leaf dry mass/total leaf area.

### Leaf Photon Absorption and Canopy Photon Capture

Leaf light absorptance was determined on the day of harvest using a spectroradiometer (PS300; Apogee Instruments) similar to the method of Nelson and Bugbee (2015). The absorptance spectrum was then multiplied by the spectral output of LEDs to obtain the leaf photon absorption under each spectral treatment (mole of photons absorbed by a single layer of leaf per mole of incident photons).

Canopy photon capture (mole of photons absorbed per mole of incident photons) was estimated by multiplying the fraction of canopy ground cover by the leaf photon absorption under each spectral treatment. This gave a good estimate in small canopies with only one layer of leaves. However, the canopy photon capture would be under-estimated in larger canopies that had over-lapping layers of leaves. To account for this, the number of leaf layers in each canopy at harvest was calculated as total leaf area at harvest (m^2^) divided by the projected canopy ground cover (m^2^). The projected canopy ground cover (or projected canopy leaf area) was determined from top down photos (*i.e.* percent ground cover multiplied by chamber ground area of 0.17 m^2^). Canopies of lettuce had relatively horizontal leaf orientation and typically did not form over-lapping leaves until one week prior to harvest. The projected canopy leaf area was thus used to estimate total canopy leaf area in young canopies. When leaves started to overlap, total canopy leaf area was interpolated from a regression function (exponential growth) fitted to the canopy leaf area estimated for young canopies and the final total canopy leaf area at harvest. The number of layers of leaves during the week prior to harvest was then calculated as the interpolated total canopy leaf area on each day divided by the projected canopy leaf area.

Using lettuce grown under spectral treatment RB 300 + FR 50 as an example, a single layer of leaves absorbed 0.798 moles of photons (integrated over 700 to 750 nm) per mole of incident photons (**Table 1**). Photon absorption with more than one layer of leaves was estimated using the Beer–Lambert equation: canopy photon absorption = 1 − e^− (k × LAI)^; where the extinction coefficient (k = 1.6) was derived by setting the photon absorption to 0.798 and LAI to 1. Thus, with two layers of overlapping leaves photon absorption would approximately equal to 1 − e^−(1.6 × 2)^ = 0.959 mol of photons absorbed per mole of incident photons. With an average of 2.6 layers of leaves at harvest, photon absorption in areas covered by leaves was adjusted to 1 − e^−(1.6 × 2.6)^ = 0.984 mol of photons absorbed per mole of incident photons.

### Canopy Quantum Yield

Canopy quantum yield for CO_2_ fixation (moles of CO_2_ fixed in gross photosynthesis per mole of photons absorbed from 400 to 750 nm) was calculated as:

(4) $${\text{Canopy quantum yield = P}}_{\text{gross}}/\text{canopy absorbed photons}$$

Note that P_gross_ here is expressed in units of moles of CO_2_ fixed per mole of incident photons for the simplicity of the equation. To convert P_gross_ from g d^−1^ in Eq. (1) to mol_CO2_ mol^−1^_incident photon_, the P_gross_ values in g d^−1^ need to be first divided by 30 g mol^−1^ (grams of dry mass per mole of carbon assimilated), then divided by 0.17 m^2^ (chamber ground area), and then divided by17.64 mol _incident photons_ m^−2^ d^−1^ (total daily photon flux density at canopy level).

### Statistical Analysis

Data were analyzed using regression (linear and sigmoid) in Sigmaplot (Systat Software, San Jose, CA) and ANOVA in Statistical Analysis Systems (SAS Institute, Cary, NC, USA). Mean separation was performed using Fisher’s protected least significant difference (LSD, *P* = 0.05). Canopy gas exchange measurements from two representative replicate studies were included in data analysis. Growth parameters and chlorophyll content were obtained from all five replicate studies.

## Results

### Biomass, Leaf Area, and Chlorophyll Content

Lettuce grown under the two control treatments without far-red (white 350 and RB 350) had nearly identical dry mass and leaf area at harvest. In contrast, both treatments with 15% FR substitution, either with a white or RB background light, had 29 to 31% increase in total biomass (shoot and root) and a 36–43% increase in leaf area (**Table 1**). Leaves of plants grown with far-red were thinner with a 4–10% decrease in leaf mass per area, ~20% lower chlorophyll content, and less efficient absorption of the incident photon on single leaf basis (**Table 1**). Dry mass partitioning to the roots, indicated by % root, did not differ significantly among the four spectral treatments even though it tended to be slightly reduced in the two treatments with FR (**Table 1**).

### Canopy Gas Exchange and Carbon Use Efficiency

**Figure 2:**
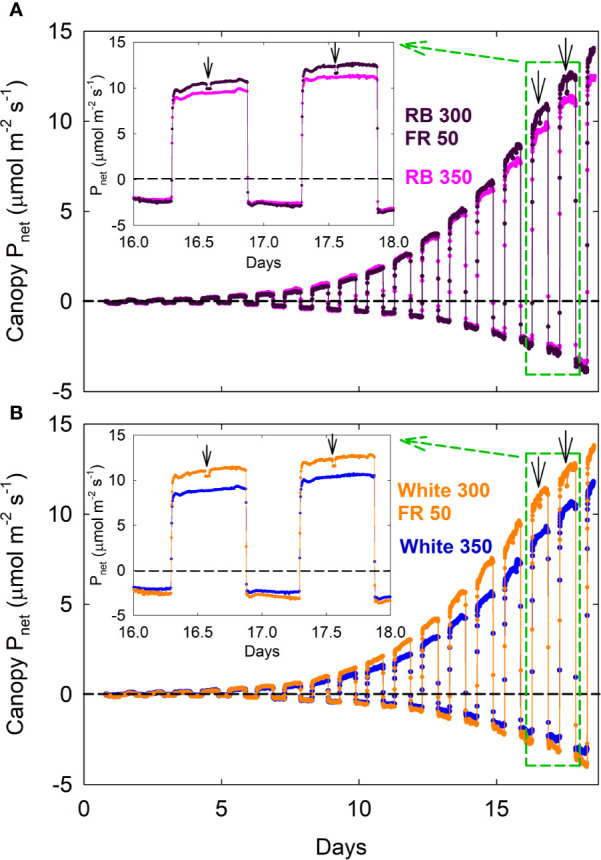
Canopy net photosynthetic rate [P_net_; net gas exchange rates in light (positive) and dark (negative) expressed as μmol_CO2_ m^−2^_ground area_ s^−1^] of lettuce under red/blue (RB) and white, with or without far-red substitution. The numbers following each type of light (*e.g.* RB 350) indicate photon flux density in μmol m^−2^ s^−1^. Plants were seeded in a greenhouse and moved into a multi-chamber gas exchange system on day zero, four days after seedling emergence. Downward pointing arrows indicate when far-red LEDs were turned off. This representative dataset of P_net_ was used to calculate daily carbon gain shown in Rep 1 of **Figure 3** carbon use efficiency in **Figure 4**, and canopy quantum yield in **Figure 7**.

**Figure 3:**
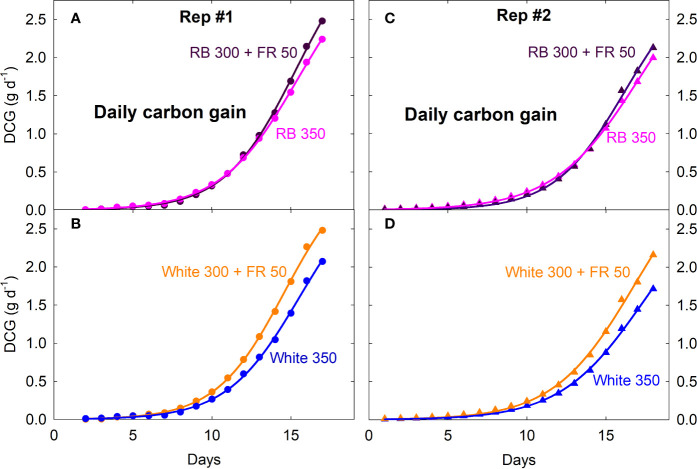
Daily carbon gain (DCG, a measure of canopy growth rate) of lettuce under four spectral treatments from seedling to mature plants. RB, red and blue; FR, far-red. The numbers following each type of light (*e.g.* RB 350) indicate photon flux density in μmol m^−2^ s^−1^. Plants were moved into the gas exchange system from a greenhouse on day zero (four days after emergence in Rep 1 and two days after emergence in Rep 2).

Canopy P_net, light_ and R_dark_ under all four spectral treatments increased exponentially from seedling to young plant stage over the first two weeks, followed by slower increases during the third week as plants quickly approached canopy closure (**Figure 2**; also see **Supplementary Figure S1** for additional dataset). Gas exchange rates were identical among all four treatments when uniformly sized seedlings were first moved into the chambers and remained similar between the white 350 and RB 350 treatments throughout the entire course of the study. Plants grown under the two FR substitution treatments, however, gradually showed higher P_net, light_ and R_dark_ (**Figure 2**) and gained more carbon per day than the control treatments (**Figure 3**). These measurements were consistent with the differences in plant dry mass under the four treatments. At harvest, dry mass predicted from the cumulative daily carbon gain obtained from gas exchange measurement was 103.9 ± 3.4% of the measured total dry mass in two representative replicate studies.

A decrease in P_net, light_ was detected within 5 min (note that each chamber was sampled every 4 min and 40 s) when the far-red LEDs in the two FR substitution treatments were turned off (**Figure 2**; insets), indicating that far-red photons directly contribute to canopy photosynthesis.

**Figure 4:**
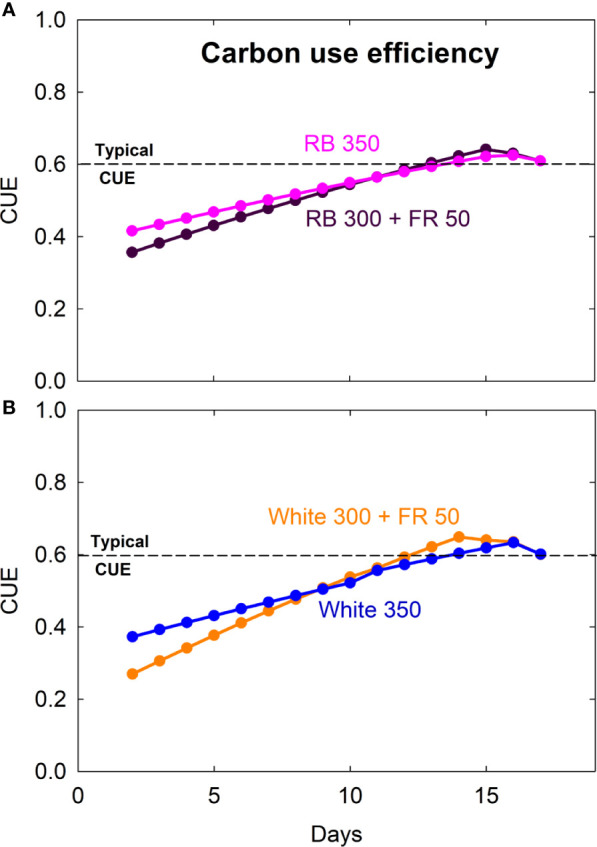
Carbon use efficiency (CUE = daily carbon gain ÷ gross photosynthesis) of lettuce grown under red/blue (RB) and white light, with or without far-red (FR) substitution. The numbers following each type of light (*e.g.* RB 350) indicate photon flux density in μmol m^−2^ s^−1^. Dashed lines indicate a typical CUE value of 0.6 that has been reported for mature plants.

Carbon use efficiency (CUE) was lower for seedlings and gradually increased as plants matured (**Figure 4**). CUE for mature plants was similar under all four treatments, with an average of 0.61 ± 0.02 (SD) over the last six days prior to harvest.

### Ground Cover and Canopy Photon Capture

**Figure 5:**
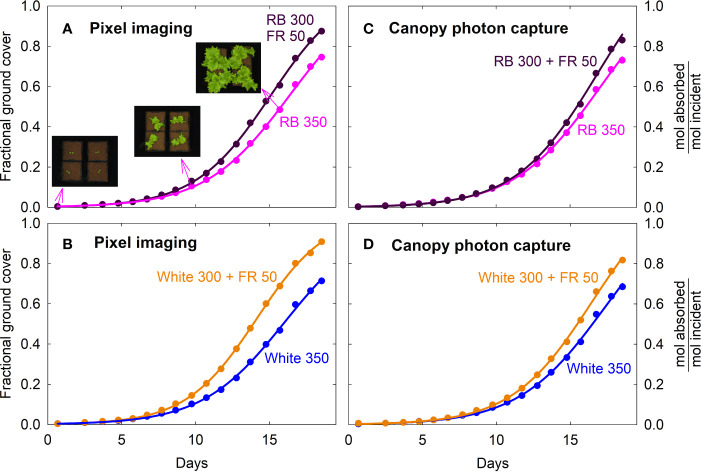
Fraction of ground cover and canopy photon capture of lettuce under four spectral treatments. Fraction of ground cover was obtained from green pixel analysis of top down photos of the canopies. Canopy photon capture (moles of photon absorbed from 400 to 750 nm per mole of incident photons) was estimated from ground cover, leaf photon absorption of the incident spectra and leaf area index. RB, red and blue; FR, far-red. The numbers following each type of light (*e.g.* RB 350) indicate photon flux density in μmol m^−2^ s^−1^.

Fractional ground cover and canopy photon capture increased over time in a similar manner as gas exchange rates and daily carbon gain (**Figure 5**; also see **Figures 2**, **3**). The FR substitution treatments consistently had higher fractional ground cover than the control treatments, especially after one week into the study (**Figures 5A, B**).

**Figure 6:**
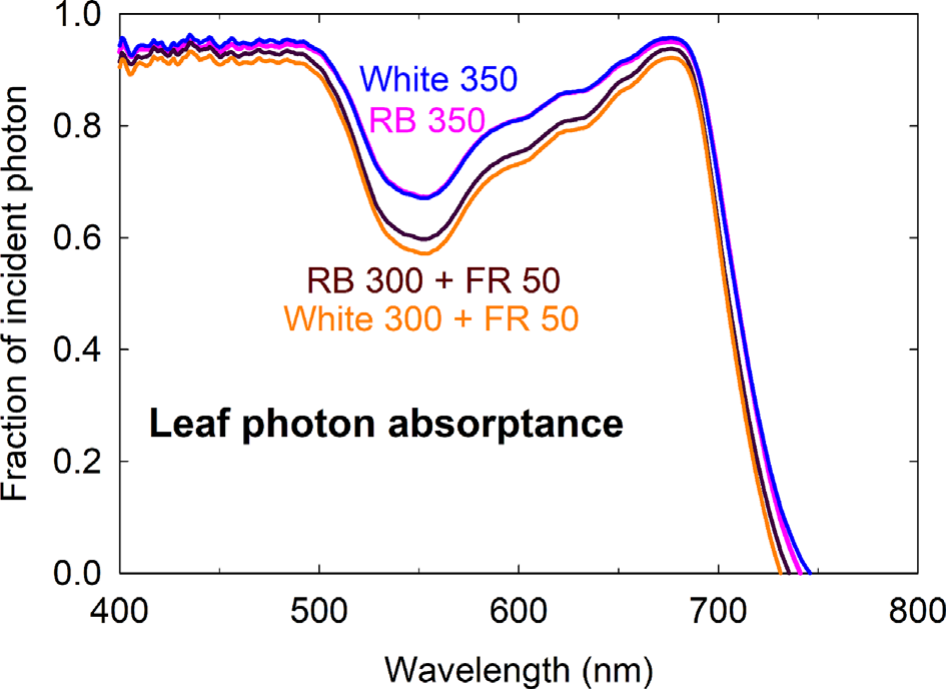
Leaf photon absorptance of lettuce grown under four spectral treatments. RB, red and blue; FR, far-red. The numbers following each type of light (*e.g.* RB 350) indicate photon flux density in μmol m^−2^ s^−1^.

The higher fractional ground cover translated into higher canopy photon capture in the FR substitution treatments, although to a smaller extent (**Figures 5C, D**). This was because 1) leaves of plants grown with far-red were thinner with lower chlorophyll content and less efficient photon absorption especially in the green and far-red regions, and 2) far-red photons in the 15% FR substitution treatments were less efficiently absorbed than the 400 to 700 nm photons in the control treatments (**Figure 6** and **Table 1**). Leaf photon absorption under each of the four light treatments was shown in **Table 1**.

### Canopy Quantum Yield

**Figure 7:**
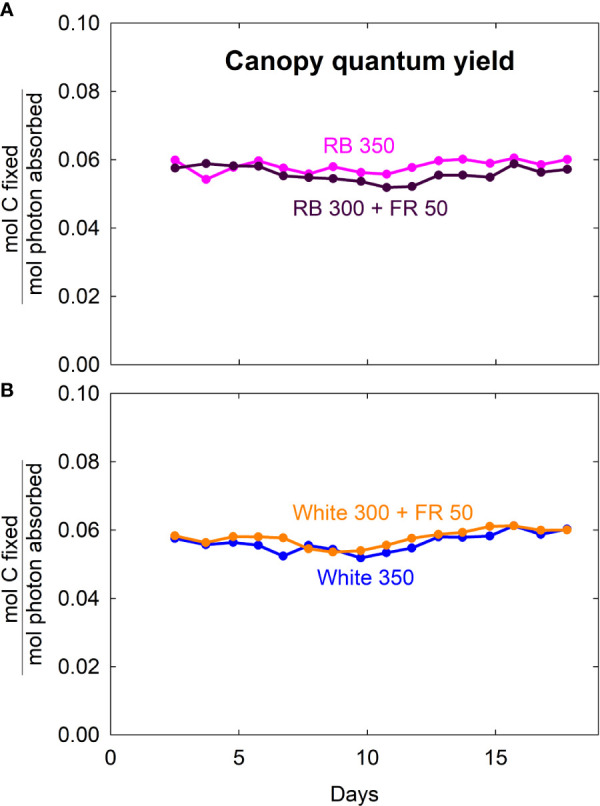
Canopy quantum yield (moles of carbon fixed in gross photosynthesis per mole of photons absorbed from 400 to 750 nm) of lettuce grown under red/blue (RB) and white light, with or without far-red (FR) substitution. The numbers following each type of light (*e.g.* RB 350) indicate photon flux density in μmol m^−2^ s^−1^.

Canopy quantum yield (moles of CO_2_ fixed in gross photosynthesis per mole of absorbed photons integrated over 400 to 750 nm) was similar among all four light treatments, with an average of 0.057 ± 0.002 (SD) (**Figure 7**). This indicates that the absorbed far-red photons were as efficient as red/blue and white photons.

### Correlation Between Canopy Photosynthesis and Photon Capture

**Figure 8:**
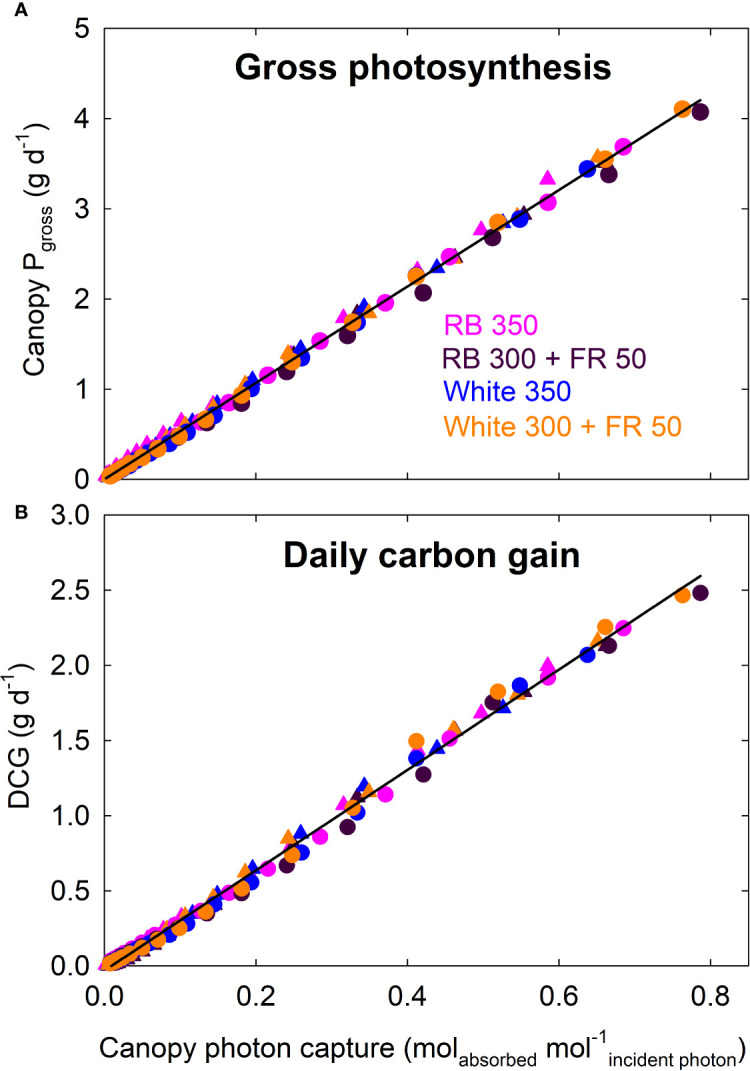
Canopy gross photosynthesis (P_gross_) and daily carbon gain (DCG) as a function of canopy photon capture of lettuce. Canopy P_gross_ (and DCG) of all four treatments increased linearly with increasing photon capture and had a common slope. RB, red and blue; FR, far-red. The numbers following each type of light (*e.g.* RB 350) indicate photon flux density in μmol m^−2^ s^−1^. Circles represent data from Rep 1, and triangles represent data from Rep 2 (see DCG in **Figure 3**).

Canopy P_gross_ of all four treatments increased linearly with increasing photon capture and had a common slope; daily carbon gain of all four treatments also showed a common linear correlation with canopy photon capture (**Figure 8**).

## Discussion

Beneficial leaf expansion elicited by far-red has been increasingly explored as means to improve production efficiency of high-value leafy green vegetables and ornamentals in greenhouses and indoor vertical farms (Goins et al., 2001; Li and Kubota, 2009; Stutte et al., 2009; Park and Runkle, 2017; Meng et al., 2019). Most of the previous studies added far-red photons as a supplement to photosynthetic photons within 400–700 nm, and the enhanced plant growth has been, in general, attributed solely to increased radiation capture through leaf expansion.

Far-red supplementation comes with an energy cost and it remains unclear whether it is cost effective to add far-red photons or to increase the traditionally defined photosynthetic photons (400 to 700 nm) by the same amount. Far-red photons contain lower energy and thus can be generated with higher efficacy (moles of photons per unit of electric energy) than red, blue, and green photons using current LED technology (Kusuma et al., 2020). Recent research on the photosynthetic effect of far-red photons has provided compelling evidence that far-red photons (700–750 nm) significantly increase leaf and canopy photosynthesis when added to photons within 400 to 700 nm (Zhen and Bugbee, 2020 and citations therein). These results indicate that far-red photons should be substituted for 400 to 700 nm photons instead of supplemented. Substitution facilitates the separation of the direct effect of far-red photons on canopy photosynthesis (by comparing the canopy quantum yield in treatments with and without far-red substitution) from their indirect effect on plant growth through altering canopy morphology and radiation capture. This provides a mechanistic understanding of the effect of far-red photons on plant growth during long-term crop cultivation.

### Leaf Expansion and Phytochrome Photoequilibrium

The increased leaf expansion in the far-red substitution treatments was presumably caused by a decrease in phytochrome photoequilibria (PPE) compared to the control treatments (white or red/blue light) (**Table 1**). PPE has been widely used to estimate the fraction of phytochromes in the active P_fr_ form relative to the total phytochrome pool (Sager et al., 1988). An inverse linear response between stem elongation rate PPE has been well-established in shade-avoiding species (Morgan and Smith, 1976; Morgan and Smith, 1979) but the relationship between leaf expansion and PPE appears to be species-specific (Smith and Whitelam, 1997; Gommers et al., 2013). The reverse correlation between leaf expansion and PPE in this study is similar to the findings by Park and Runkle (2017). However, the slope of the relationship varied with the background light (**Supplementary Figure S2**). It is noteworthy that the total canopy leaf area was nearly identical in both treatments with far-red substitution, despite that the estimated PPE differed greatly (0.70 under white *versus* 0.82 under red/blue background light). Similarly, canopy leaf area was nearly identical in the two control treatments in spite of the variation in PPE: 0.84 under white and 0.87 under red/blue light.

Phytochrome photoequilibria cannot be directly determined in green leaves due to the masking effect of chlorophylls (which also fluoresce) (Siegelman and Butler, 1965; Holmes and Fukshansky, 1979). Instead, PPE has been estimated from the spectral distribution of incident light and the photoconversion coefficients of phytochrome P_r_ and P_fr_ that are purified from etiolated tissue (Sager et al., 1988). It has been recognized that PPE estimated based on this approach may not consistently predict plant responses. Contributing factors for the variability were reviewed by Rajapakse and Kelly (1994). Among which, the incident light that reaches phytochrome is filtered by chlorophylls, thus PPE estimated from incident light spectrum often do not reflect PPE within different layers of cells within a leaf, nor in leaves from different positions within a canopy (Morgan and Smith, 1978; Jose and Schäfer, 1978; Holmes and Fukshansky, 1979). The white LED light in this study contained a large fraction of green photons and a much smaller fraction of red photons compared to the red/blue light (**Figure 1**). The absorption spectrum of phytochrome ranges from 300 to 800 nm (Sager et al., 1988). Because chlorophylls more efficiently absorb red photons than green photons, the extent of chlorophyll masking effect on the phytochrome response most likely differed under the white and red/blue light. This may be responsible for the different relationships between leaf expansion and estimated PPE under white and red/blue light.

### Leaf Light Absorptance, Canopy Ground Cover, and Radiation Capture

The increased yield in response to FR supplementation is often attributed to increased photon capture (Goins et al., 2001; Li and Kubota, 2009; Stutte et al., 2009; Park and Runkle, 2017), but canopy photon capture is rarely quantified. Most studies report only leaf area at harvest, which non-linearly correlates with canopy photon capture due to overlapping of leaves. Goins et al. (2001) reported that lettuce grown with 12% added far-red photons consistently had higher canopy ground cover over a two-week growing period than plants that received equal photon flux in the PAR region. As expected, the increased leaf expansion under the far-red substitution treatments led to greater fractional canopy ground cover (**Figures 5A, B**). Fractional ground cover was found to be highly correlated with canopy photon absorption (r^2^ = 0.99) of lettuce (Klassen et al., 2004), but the relationship was nonlinear and varied over time. Other factors, including leaf optical properties and the spectral distribution of incident light, can significantly alter spectral effects on canopy photon capture.

Leaf thickness and chlorophyll content affect leaf optical properties (Vogelmann, 1993). Reduced leaf chlorophyll concentration is a nearly universal response to FR irradiation among a wide range of species, regardless of changes in leaf expansion (Morgan and Smith, 1979; Casal et al., 1987; Smith and Whitelam, 1997; Li and Kubota, 2009; Meng et al., 2019). Several causes for this decrease in chlorophyll content have been suggested, including 1) limited photosynthate supply in shade-avoiding species that had reduced leaf expansion, 2) a dilution effect when leaf expansion is promoted, and 3) a direct effect on chlorophyll biosynthesis due to reduced amount of active P_fr_ relative to the total pool of phytochromes (see Casal et al., 1987 and references therein). The stimulatory effect of far-red photons on leaf expansion has likely contributed to the ‘dilution’ of chlorophyll content per unit leaf area. The reduction in leaf chlorophyll content and thickness (indicated by the lower leaf mass per area in **Table 1**) of lettuce grown with far-red substitution resulted in low leaf light absorptance, especially in the green and far-red regions of the spectrum (**Figure 6**).

In addition, the far-red photons were less efficiently absorbed by the leaves, which reduced canopy photon capture (**Table 1**). As a result, the magnitude of increase in canopy photon capture in the FR substitution treatments was smaller than indicated by the fractional ground cover. Therefore, the use of total leaf area or canopy ground cover would likely lead to over-estimation of canopy photon capture.

### Predicted Dry Mass From Gas Exchange Measurements

All gas exchange systems measure the carbon assimilation rate in moles of carbon (CO_2_) fixed per unit time. To convert P_gross_ and DCG from moles of carbon to grams of biomass, we assumed that the canopy carbon content of lettuce was 0.4 g _carbon_ g^−1^_dry mass_. Carbon fraction in plants has often been assumed to be 43 to 45% (Epstein and Bloom, 2005), but this fraction varies widely with the composition of the biomass (carbohydrates, lignin, proteins, lipids; Amthor, 2010). The carbon fraction thus varies with type of vegetation (leaves, stems and roots) and species (Vertregt and Penning de Vries, 1987; Monje and Bugbee, 1998). It also varies during ontogeny (Ho, 1976; Hadley and Causton, 1984).

Growing conditions also affect carbon fraction. Becker et al. (2015) found that the carbon fraction in leaves of green lettuce decreased from 0.4 to 0.38 g g^−1^ as nitrogen availability increased. Monje and Bugbee (1998) reported that leaf carbon content of wheat increased from 0.38 under ambient CO_2_ to 0.4 g g^−1^ under elevated CO_2._ These changes were small but reproducible. Lettuce is about 85% leaves (**Table 1**). Roots tend to have a higher carbon content than leaves (0.44 to 0.45 g g^−1^ in roots of wheat; Monje and Bugbee, 1998). The weighted carbon content of the combined leaves and roots of the plants in these studies in thus estimated to be 0.4 g g^−1^. With this value, the dry mass at harvest predicted from the cumulative DCG averaged 103.9% of the measured total dry mass. This close match validates the accuracy of the gas exchange measurements.

### Canopy Quantum Yield: Short-Term *Versus* Long-Term Response to Far-Red Photons

Measurements of canopy gas exchange rate coupled with canopy photon absorption enable the calculation of canopy quantum yield, a measure of photosynthetic efficiency expressed as moles of CO_2_ fixed in gross photosynthesis per mole of photons absorbed. We calculated quantum yield using wavelengths from 400 to 750 nm. To our knowledge, this is the first report of the long-term effect of far-red photons on canopy quantum yield. The lack of difference in canopy quantum yield among the four spectral treatments indicates that the absorbed far-red photons were equally efficient as red/blue or white photons (**Figure 7**). This is also indicated by the nearly identical relationship between canopy P_gross_ and photon capture among the treatments (**Figure 8A**).

On an incident photon flux basis, Zhen and Bugbee (2020) showed that the short-term (hours) response of canopy photosynthesis to far-red photons from 700 to 750 nm was equal to 400 to 700 nm photons when applied simultaneously. Because far-red photons are less efficiently absorbed than 400 to 700 nm photons, the far-red photons had a higher canopy quantum yield on an absorbed photon basis. This difference between short-term and long-term responses indicates that the efficiency of light utilization for photosynthesis may have acclimated to far-red radiation during long-term growth. Possible changes include modifications in leaf thickness, chlorophyll content (**Table 1**), chl a:b ratio (Gommers et al., 2013; Kalaitzoglou et al., 2019), PSI:PSII ratio, electron transport and photosynthetic capacity, rubisco content and activity (Chow et al., 1990a; Chow et al., 1990b; Anderson et al., 1995), as well as in the biosynthesis of photoprotective pigments carotenoids and anthocyanins (Li and Kubota, 2009; Stutte et al., 2009; Kalaitzoglou et al., 2019).

### Carbon Use Efficiency

Respiration is often assumed to be a fixed fraction of gross photosynthesis in modeling maximum productivity per unit input of solar energy (Zhu et al., 2008; Zhu et al., 2010; Amthor, 2010). A constant CUE of ~0.5 has been widely used in forest growth models (Waring et al., 1998; DeLucia et al., 2007). Gifford (1994) reported that the ratio of respiration to gross photosynthesis of whole plants (which equals to 1—CUE) was constant around 0.4 (a CUE of ~0.6) in diverse annual and perennial species and was minimally affected by plant age and growth temperature (15 to 30°C). Monje and Bugbee (1998) found that CUE of wheat grown under near-optimal conditions (high light and ample water and nutrients) was constant around 0.6 (except during the first week after emergence and final week when plants senesced) despite changes in carbon partitioning to leaves, stems, and seeds. The response was not affected by [CO_2_] even though canopy quantum yield and biomass production increased significantly when [CO_2_] was elevated to 1200 mol mol^−1^ (Monje and Bugbee, 1998).

However, reports have conflicted on whether CUE stays constant among species, environmental conditions, and developmental stages (Amthor, 2000; Cannell and Thornley, 2000). A constant CUE means that plants always respire the same fraction of carbohydrates regardless of growth rate. To better understand the link between respiration and growth, respiration is frequently divided into two functional components—*growth respiration* that is determined by the synthesis of new biomass (proportional to growth rate) and *maintenance respiration* that keeps existing biomass functional (proportional to plant size) (McCree, 1970; Amthor, 1989; Amthor, 2000). The respiration required per unit of new growth (*i.e.* growth respiration coefficient) depends on the chemical composition of the biomass being synthesized (Penning de Vries et al., 1974; Amthor, 2010). The respiration required to maintain existing biomass per unit time (*i.e.* maintenance respiration coefficient) is affected by metabolic rate (associated with temperature), but it can also be influenced by the chemical composition, especially tissue nitrogen content (Cannell and Thornley, 2000). van Iersel (2003) showed that CUE can be expressed as function of relative growth rate and growth and maintenance respiration coefficients. Because of the ontogenetic changes in plant tissue composition and relative growth rate, a constant CUE is unlikely unless growth and/or maintenance respiration coefficient change concurrently with relative growth rate. van Iersel (2003) further demonstrated that CUE of lettuce decreased from 0.6 in young plants (24-day old) to 0.2 in old plants (66-day old). This was mainly due to the decrease in relative growth rate and increase in the fraction of maintenance respiration with increasing plant size. The carbon use efficiency of fast-growing and compositionally simple crops like lettuce is expected to be relatively more constant under optimal conditions, where a higher relative growth rate would reduce the importance of maintenance respiration (van Iersel, 2003). Frantz et al. (2004) found that the CUE of rapidly growing lettuce (~15 to 35 day old) under optimal conditions (high light, elevated CO_2_, and a constant day/night temperature of 25°C) was stable around 0.62. Decreasing night temperature from 34 to 17°C reduced nighttime respiration rate and allocation to shoot/root, but only slightly altered CUE.

In this study, CUE of young/newly matured lettuce plants (~16–22 days after emergence) was similar under all four treatments, with an average of 0.61 ± 0.02. This is consistent with the values reported for lettuce of similar age by van Iersel (2003) and Frantz et al. (2004). However, CUE of younger seedlings was not determined in the previous studies because of the difficulties in obtaining accurate gas exchange measurements with small plants. With an improved gas-exchange system, we observed that CUE of young lettuce seedlings gradually increased within the first 2 weeks after emergence, similar to the gradual increase in CUE of wheat during the first 8 days after emergence (Monje and Bugbee, 1998). A gradual increase in CUE was also reported in young vinca seedlings as the ratio of respiration to photosynthesis decreased during the first few weeks of plant development (van Iersel and Seymour, 2000). The similarity in CUE of young/newly matured lettuce plants among the spectral treatments indicates that the phenotypical changes caused by FR radiation had little effect on CUE.

## Concluding Remarks

Overall, we found that neither canopy quantum yield nor carbon use efficiency were affected when substituting far-red for traditionally defined photosynthetic photons. This indicates that the absorbed far-red photons (700–750 nm) were equally efficient for photosynthesis when acting synergistically with 400–700 nm photons. Crop yield increased with far-red substitution due to increased leaf expansion and canopy radiation capture mediated by phytochromes during long-term plant cultivation. These data, coupled with previous studies, provide compelling evidence that the current definition of photosynthetically active radiation should be extended to include photons from 700 to 750 nm.

## Data Availability Statement

All datasets presented in this study are included in the article/**Supplementary Material**.

## Author Contributions

SZ and BB designed the experiment. SZ performed the experiment, analyzed data, and wrote the first draft. SZ and BB discussed the data and revised the manuscript.

## Funding

This work was supported by the Utah Agricultural Experiment Station, Utah State University; by the NASA-CUBES project award number NNX17AJ31G; and by the USDA-NIFA-SCRI award number 2018-51181-28365 (LAMP Project). Any opinions, findings, and conclusions or recommendations expressed in this material are those of the authors and do not necessarily reflect the views of the National Aeronautics and Space Administration (NASA) or the USDA.

## Conflict of Interest

The authors declare that the research was conducted in the absence of any commercial or financial relationships that could be construed as a potential conflict of interest.

## Supplementary Material

Supplementary Figure 1 Canopy net photosynthetic rate (P_net_; μmol_CO2_ m^−2^_ground area_ s^−1^) of lettuce under red/blue (RB) and white, with or without far-red substitution. The numbers following each type of light (*e.g.* RB 350) indicate photon flux density in μmol m^−2^ s^−1^. Plants were seeded in a greenhouse and moved into a multi-chamber gas exchange system two days after seedling emergence (indicated by Day 0 on the x-axis). Downward pointing arrows indicate when far-red LEDs were turned off.

Supplementary Figure 2 Relationships between estimated phytochrome photoequilibria (PPE) and leaf area of lettuce grown with or without far-red photons under two background light spectra—white and red/blue (RB). The decrease in the estimated PPE under each background light was due to a 15% far-red substitution. All four spectral treatments had equal total photon flux of 350 μmol m^−2^ s^−1^ (400 to 750 nm) and 31.5% blue photons.

## Abbreviations

CUE, carbon use efficiency; DCG, daily carbon gain; LAI, leaf area index, LED, light emitting diode; Canopy P_gross_ and P_net_, canopy gross and net photosynthetic rate; PAR, photosynthetically active radiation from 400 to 700 nm; PPFD, photosynthetic photon flux density integrated over 400 to 700 nm; PFD, photon flux density; R_dark_, dark respiration.
